# The *daf-7(e1372)* mutation rescues dauer formation defects seen in *C. elegans unc-33* mutants

**DOI:** 10.3389/fphys.2023.975878

**Published:** 2023-02-06

**Authors:** Alexia Samaro, Alejandra Cristancho, Alexis Rivas, Ruby Valtierra, Skye Beck, Jason Cantu, Maria Miranda, Arianna Vacio, Oscar Cardenas Muedano, Andrea Holgado

**Affiliations:** Department of Biological Sciences, St. Edward’s University, Austin, TX, United States

**Keywords:** dauer, TGF-β, UNC-33, *C. elegans*, locomotion, axonal outgrowth

## Abstract

Collapsin response mediator protein-2 (CRMP2) in humans, UNC-33 in *C. elegans*, is a molecule that mediates axonal outgrowth and stability. UNC-33/CRMP2 has been hypothesized as a potential drug target for treating Alzheimer’s and other neurodegenerative diseases, which can often be attributed in part to aging. In aging, CRMP2 becomes hyperphosphorylated, which decreases the protein’s functionality, destabilizes the cellular skeleton, and contributes to neurodegeneration. In *C. elegans,* aging can be slowed by entering dauer diapause; a non-aging developmental stage turned on when the DAF-7/TGFβ signaling pathway is silenced in response to environmental stressors. In our laboratory, we discovered that *unc-33* mutants are unable to form dauers in response to environmental stressors, but the mechanism behind this is still unknown. Here, we present a study that investigates whether a mutation in the *daf-7* gene which leads to a temperature sensitive constitutive dauer phenotype can rescue phenotypes characteristic of *unc-33* mutants. To this end, we created *unc-33*; *daf-7* double mutants and quantified proper dauer formation after exposure to unfavorable environmental conditions. In addition, we tested how the introduction of the *daf-7* mutation would affect the locomotion of the double mutants on an agar plate and a liquid medium. Furthermore, we examined axonal elongation of the double mutants using a transgene, *juIs76,* which expresses GFP in GABAergic motor neurons. Our analysis of *unc-33; daf-7* double mutants showed that introducing the *daf-7* mutation into an *unc-33* mutant rescued dauer formation. However, further studies revealed that the *unc-33; daf-7* double mutants had defects in axonal outgrowth of their D-type motor neuron which had been previously seen in *unc-33* single mutants and impaired locomotion. Based on these results, we concluded that *unc-33* mutants might have a problem suppressing DAF-7 signaling under unfavorable environmental conditions, leading to the activation of reproductive programs and the development of adults instead of dauers.

## 1 Introduction

The *unc-33* gene in *Caenorhabditis elegans* (*C. elegans*) is the ortholog of the human collapsin response mediator protein 2 or CRMP2/DPYSL2. This gene encodes for three different isoforms through alternative splicing, which are UNC-33S, UNC-33M, and UNC-33L (Tsuboi et al., 2005). In vertebrates, CRMP2 is vital for the polarity of neurons, growth of axons, strength of the synapse, neuronal migration and differentiation, release of neurotransmitters, and survival of neurons (Na et al., 2017). Hyperphosphorylation and inactivation of CRMP2 has been detected in many neurological disorders, including Alzheimer’s and Parkinson’s disease, ALS, and chronic pain, and could thus be a promising drug target for the treatment of neurological diseases (Williamson et al., 2011; Moutal et al., 2019; Togashi et al., 2019). As for *C. elegans*, UNC-33 is fundamental for the growth and guidance of the axons in sensory neurons, interneurons, and motor neurons. Research conducted by Maniar and colleagues has shown that UNC-33 has a primary role in supporting the asymmetry of the two different compartments in neurons, axons, and dendrites (Mainar et al., 2011). UNC-33 is also needed for normal body movements, laying eggs, and defecation. Characterization of various *unc-33* mutations resulted in identifying null or hypomorph mutants. The null mutant, allele *mn407,* possesses a 500 base pair deletion resulting in an early stop codon and no *unc-33* gene products, but is viable and fertile (Li et al., 1992; Tsuboi et al., 2005). The hypomorph mutant, allele *e204,* has a single nucleotide change that produces the substitution of a conserved Asp amino acid to Asn (D 389 N) (Li et al., 1992; Tsuboi et al., 2005). Studies have found that *unc-33(e204)* mutants mislocalize UNC-33 proteins to the cell body of neurons instead of the ventral nerve cord and nerve ring where the proteins normally accumulate (Tsuboi et al., 2005). As a result, *unc-33* mutants have numerous axonal outgrowth defects, suggesting that the localization of UNC-33 is vital for the outgrowth of axons (Li et al., 1992; Tsuboi et al., 2005). Furthermore, both *unc-33(e204)* and *unc-33(mn407)* mutants are short and dumpy, exhibit uncoordinated locomotion, and have defects in axonal guidance of sensory and motor neurons (Brenner et al., 1974; Hedgecock et al., 1985; Li et al., 1992).

More recently, studies reported that *unc-33* mutants are incapable of forming dauers (Lopez et al., 2022). The formation of dauers is an alternative developmental non-aging stage in the *C. elegans* life cycle that is induced by harsh conditions sensed by larvae at the L1 life stage. The persistence of these environmental conditions leads to the formation of an L2d followed by the entry into dauer diapause (Karp, 2018). *C. elegans* entry into the dauer diapause is mainly regulated by two different pathways: the insulin/DAF-2 signaling cascade and the TGFβ/DAF-7 signaling pathway (Fielenbach and Antebi, 2008). Upon inactivating of the DAF-2 or DAF-7 pathways, mTOR is repressed, autophagy is induced, and L1, L2 respond by becoming dauers (Fielenbach and Antebi, 2008). DAF-7 is the *C. elegans* homolog of transforming growth factor beta (TGFβ). The TGFβ signaling pathway enables processes such as development, regulation of the immune system, and differentiation of adult stem cells in humans (Wang and Levy, 2006). In the case of *C. elegans*, the DAF-7 signaling pathway regulates reproductive development, and when disabled, it leads to dauer diapause. The expression of *daf-7* is enriched in ASI ciliated neurons, and the secretion of DAF-7 is regulated by environmental signals (Wang and Levy, 2006). In favorable environmental conditions, DAF-7 is secreted from ASI neurons. This secreted factor binds to DAF-1/4 serine-threonine kinase receptors, which then phosphorylate DAF-8 and DAF-14, translocating them to the nucleus, inhibiting DAF-3 and DAF-5, leading to the development of adults. In unfavorable environmental conditions, DAF-7 is not secreted, allowing for the formation of dauers (Fielenbach and Antebi, 2008). Moreover, mutations in the genes that regulate dauer formation can either result in a defective dauer formation phenotype or constitutive dauer formation phenotype (Murakami et al., 2001). As such, the *daf-7(e1372*) point mutation results in a temperature sensitive phenotype producing 100% dauers at 25°C (Ren et al., 1996).

To better understand the inability of *unc-33* mutants to enter dauer diapause in response to environmental stress, we created two double mutants, *unc-33 (mn407); daf-7 (e1372)* and *unc-33(e204); daf-7(e1372),* to examine their phenotypes. First, we assessed whether the introduction of the *daf-7* mutation restores dauer formation by isolating dauers *via* a treatment with 1% SDS. Next, we characterized the locomotion and axonal outgrowth of these double mutants. Collectively, our results show that the introduction of the *daf-7(e1372)* mutation successfully restores the production of dauers in *unc-33* mutants exposed to unfavorable environmental conditions. Furthermore, we found that adding *daf-7(e1372)* mutation to an *unc-33* mutant background does not rescue the uncoordinated movements in *unc-33* mutants, nor does it rescue the axonal elongation defects.

## 2 Materials and methods

### 2.1 Strains

Strains Wild-type N2, CB204 *unc-33(e204)* IV, CB1372 *daf-7(e1372)* III, SP1382 *unc-33(mn407)* IV*,* CZ13799 *juIs76* [*unc-25p::GFP + lin-15(+)*] II*,* and DR466 *him-5* (e1490) V were provided by *Caenorhabditis* Genetics Center (CGC). Strains AMH113 *daf-7(e1372)* III*; unc-33(mn407)* IV, AMH115 *daf-7(e1372)* III*; unc-33(e204)* IV*,* AMH32 *juIs76* II; *unc-33(mn407)* IV*,* AMH34 *juIs76* II; *unc-33(e204)* IV, AMH145 *juIs76* II; *daf-7(e1372)* III; *unc-33(mn407)* IV*,* AMH151 *juIs76* II; *daf-7(e1372)* III*, AMH155 juIs76* II; *daf-7(e1372)* III; *unc-33(e204)* IV were generated in our laboratory.

All strains were grown and maintained on standard NGM agar plates at 20°C on an *Escherichia coli* OP50 lawn (Brenner, 1974). Strains containing *daf-7(e1372)* mutation were subject to incubation of 25°C to induce dauer formation. According to Ren and colleagues, *daf-7(e1372)* produce 100% dauers at 25°C and 5% at 20°C (Ren et al., 1996).

### 2.2 Genotyping

10–20 worms per strain were put into PCR tubes containing 10 µL of worm lysis buffer (10 mM Tris pH 8.2, 2.5 mM MgCl_2,_ 50 mM KCl, 0.45% NP40, 0.45% Tween 20) with 100 ug/ml of proteinase K. Tubes were then placed at −80°C for 20 min, and worm lysis was finalized by incubating tubes for 90 min at 62°C and 15 min at 95°C. Worm lysates were then combined with the appropriate primers (see table 1), PCR master mix at a final concentration of 1X (Apex Bioresearch), and placed in a thermocycler for amplification. Fragments of the *unc-33* gene were amplified using the following parameters: 5 min at 94°C, then 25 cycles of 30 s at 94°C, 30 s at 47°C, 1 min at 72 °C, 7 min at 72°C, and stored at 4°C. Fragments of the *daf-7* gene were amplified using the following parameters: 5 min at 94°C, then 25 cycles of 30 s at 94°C, 30 s at 49°C, 1 min at 72 °C, 7 min at 72°C, stored at 4°C. PCR amplicons were then evaluated using 1% agarose gel electrophoresis. After evaluation, PCR amplicons were cleaned using the QIAGEN QIAquick PCR Kit and following manufacturer recommendations. Cleaned PCR fragments were sent to be sequenced.

**TABLE 1:** Gene specific primers used for genotyping.

Gene (allele)	Primers
*unc-33(mn407)*	Forward: 5’ TGC AAA ACC TCT GAA AAA GC 3’
	Reverse: 5’ CGC AAA TCT CAA ATC CTG AC 3’
*unc-33(e204)*	Forward: 5’ TGC AAA ACC TCT GAA AAA GC 3’
	Reverse: 5’ GTT CCC ATA CGA CTG CCA TG 3’
*daf-7(e1372)*	Forward: 5’ CCG GAT TTG ACG AAA CTT TAC TAG 3’
	Reverse: 5’ GGA GAA ATT GTG AAC CAA CTG G 3’

### 2.3 1% sodium dodecyl sulfate (SDS) treatment

Twenty gravid hermaphrodites were used to synchronize nematodes from strains containing the *daf-7(e1372)* mutation. The hermaphrodites were placed on NGM agar plates containing an OP50 lawn and incubated at 25°C for 6 hours. After 6 hours, gravid hermaphrodites were removed, and the synchronous eggs were incubated at 25°C for 4 days. After 4 days, nematodes were resuspended with 1 mL DI water and transferred into 1.5 mL tubes. Worms were centrifuged for 1 min at 9,221 x g and pellet worms were treated with 1 mL of 1% SDS for 20 min at room temperature on an orbital shaker. After the 20-min incubation, worms were centrifuged for 1 min at 9,221 x g and the supernatant was removed. Pelleted worms were washed 3 times using 1 mL of M9 buffer and centrifuged for 1 min at 9,221 x g at 20°C after each wash. After the last wash, 100 uL of pellet worms were transferred onto an NGM plate with no bacteria and the percent survival was quantified (Lopez et al., 2022). Strains that did not contain the *daf-7(e1372)* mutation were grown on NGM agar plates containing a spot of OP50 at 25°C until all OP50 was consumed. Nematodes were then starved for 7 days at 25°C. After starvation, nematodes were exposed to 1% SDS treatment as described, and survival was calculated.

### 2.4 Locomotion on agar

In order to measure locomotion, an animal in the L3 or dauer stage was placed onto an NGM agar plate containing an OP50 lawn and was allowed to move for 10 min at room temperature (Zhang and Koch, 2017). The distance traveled was imaged using a Leica Stereo Microscope, and the LASX software was used to measure the distance traveled by the animal. The same protocol was followed for assessing locomotion on agar for adults that developed after exiting the dauer stage. These adults were obtained after incubating dauers at 20°C for a minimum of 2 days. Locomotion was measured as distance traveled after 10 min. The locomotion of a total of 60 hermaphrodites per strain was analyzed. dedevlop.

### 2.5 Locomotion on liquid

The motility in liquid of L3 nematodes, dauers, as well as adults that developed after exiting the L3 or the dauer stage was quantified after following these steps. First, 1 mL of deionized water was placed in each well on a 24-well plate at room temperature. Next, individual nematodes were picked from the plate and suspended in one of the wells. After a 10–15 s acclimation period, the body movements within 30 s were recorded. One isolated body movement of head, torso, or tail was considered one thrash. A total of 60 hermaphrodites per strain was quantified.

### 2.6 Axonal outgrowth

In order to assess axonal outgrowth, nematodes of the L3 stage or dauer stage were placed on an agar pad containing 5 μL of 200 mM Levamisole to immobilize them. These nematodes were then imaged using an Olympus fluoview confocal microscope at a total magnification of 600X, and z-stacks of the dorsal nerve cord were captured. Each animal’s whole dorsal nerve cord was recorded using two z-stack images, one containing the head to the midsection of the worm and the other focusing on the midsection to the tail. Once the z-stacks were captured, the images were then turned into a projection using the Olympus CellSens software, and the projections were then stitched together using a Multiple Image Alignment. Axonal outgrowth in the dorsal nerve cord was analyzed by measuring the number and length of the gaps within the dorsal nerve cord using an ROI polyline in CellSens.

### 2.7 Statistical analysis

Statistical analysis of data collected that followed a normal distribution was performed using a one-way ANOVA with Tukey’s multiple comparison *post hoc* test after testing for homogeneity of variance using the Brown-Forsythe Test. Data that did not pass the Shapiro-Wilk test for normal distribution were analyzed using Kruskal–Wallis’s test with Dunn’s multiple comparison test. All statistical analyses and graphs were generated with Prism, Grand GraphPad.

## 3 Results

### 3.1 A mutation in *daf-7*/TGFβ rescues dauer formation defects in *unc-33* mutants

**FIGURE 1:**
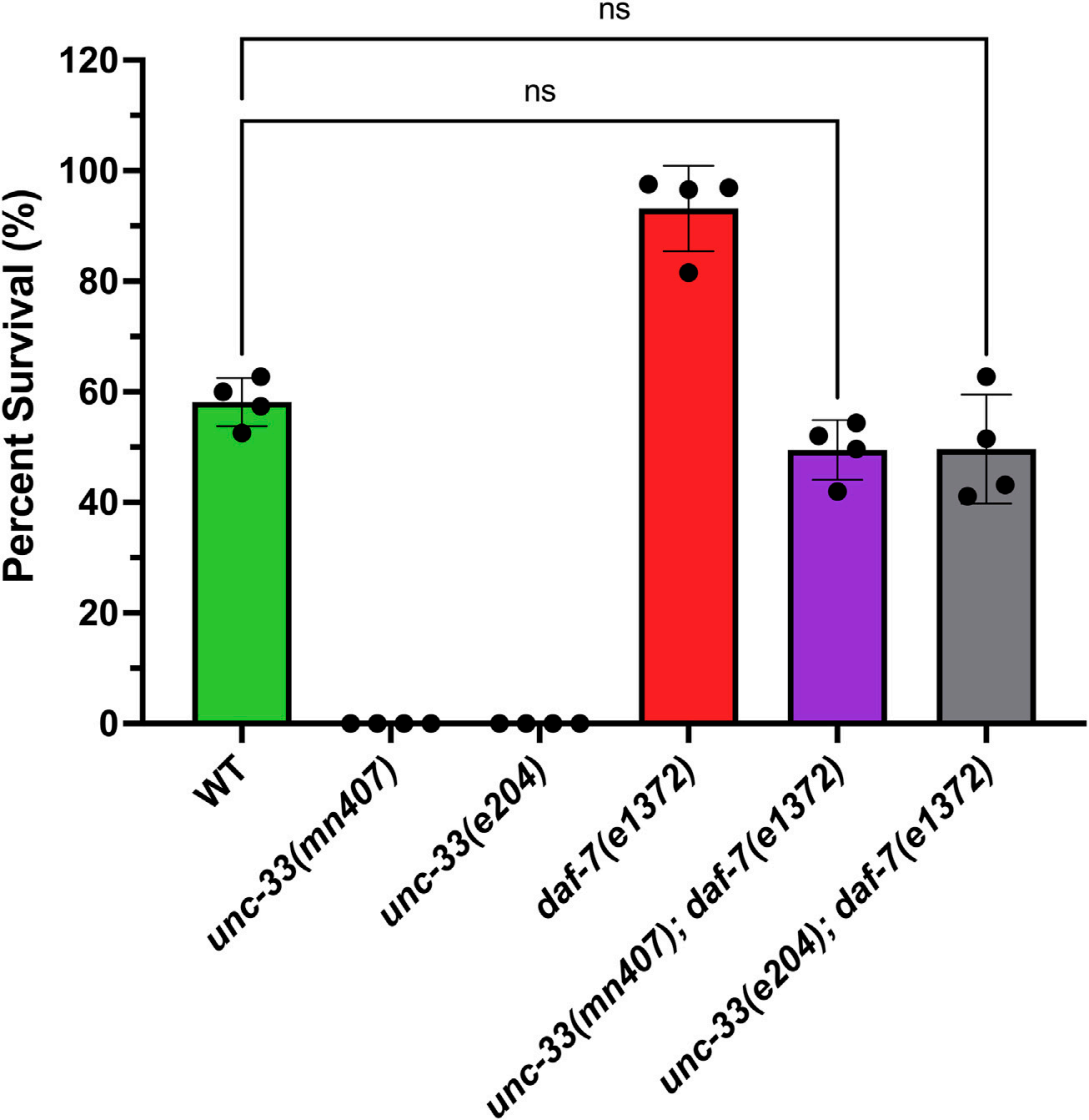
Addition of the *daf-7* mutation into *unc-33* mutants restores dauer formation. Percent survival of all strains after stimulation of dauer formation followed by 1% SDS treatment was quantified. *unc-33(mn407)* and *unc-33(e204)* had no detectable survival to 1% SDS confirming that *unc-33* mutants are incapable of forming dauers. Analysis of one-way ANOVA with Tukey’s multiple comparisons test shows that the percent survival of double mutants *unc-33; daf-7* was not statistically different from wildtype. However, *unc-33(mn407)* and *unc-33(e204)* were statistically different from wildtype (*p* < 0.0001). Plotted is the mean percent survival +/- standard deviation of 4 independent replicas per strain. ns = not significant.

Previous studies have shown that *unc-33* mutants fail to form dauers under starvation conditions (Lopez et al., 2022); however, the mechanism behind this inability is still unknown. To test the hypothesis that the inability of *unc-33* mutants to enter dauer diapause stems from an issue within the DAF-7/TGFβ pathway, strains containing the *unc-33* mutation (allele *mn407* or *e204*) and the temperature-sensitive mutation *daf-7(e1372)* were generated. Once these double mutant strains were confirmed *via* genotyping, strains grown in dauer-inducing conditions were exposed to 1% SDS treatment, and their survival was quantified. Strains with the *daf-7* mutation were synchronized and grown at 25°C, then exposed to 1% SDS treatment. The results confirmed that *unc-33* mutants die after the SDS treatment suggesting that they do not form dauers (Figure 1). Wildtype nematodes exposed to starvation for 7 days at 25°C produced 58% ± 4 dauers while *daf-7(e1372)* incubated at 25°C for 4 days formed 93% ± 8 dauers. Additionally, these results revealed that the double mutant strains *unc-33(mn407); daf-7(e1372)* and *unc-33(e204); daf-7(e1372)* form dauers as they are resistant to 1% SDS (Figure 1). One-way ANOVA with Tukey’s multiple comparisons shows that the percent survival of double mutants *unc-33(mn407); daf-7(e1372)* and *unc-33(e204); daf-7(e1372)* to 1% SDS is not significantly different from wildtype nematodes (*p* = 0.331 and *p* = 0.346, respectively). However, the percent survival of double mutants *unc-33(mn407); daf-7(e1372)* and *unc-33(e204); daf-7(e1372)* is different from *daf-7(e1372)* (*p* < 0.0001). Together, this data suggests that *unc-33* mutants could have a defect that prevents them from inhibiting the synthesis or secretion of DAF-7 in response to environmental stressors, thus failing to form dauers under these conditions.

### 3.2 Dauers *unc-33; daf-7* double mutants have uncoordinated movements

**FIGURE 2:**
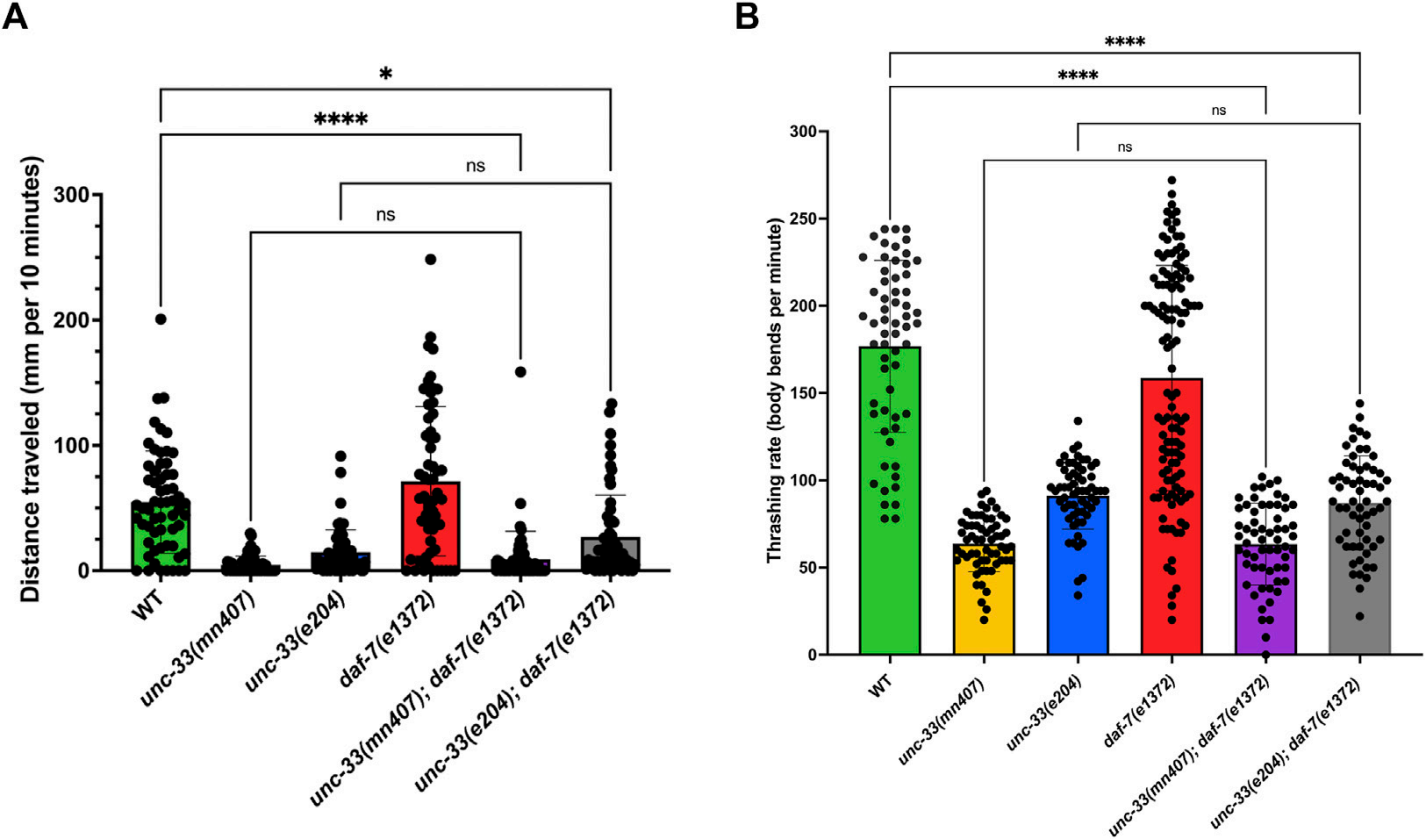
Double mutant *unc-33; daf-7* dauers have similar rates of locomotion to *unc-33* single mutant nematodes **(A)** The average distance traveled in millimeters per 10 minutes for each strain on a plate seeded with *E. coli* OP50 is plotted. **(B)** Average thrashing rate (body bends per minute) of each strain placed in liquid media. Statistical analyses using Kruskal–Wallis’s tests followed by Dunn’s multiple comparisons show no significant differences (ns) between double mutants *unc-33; daf-7* and their *unc-33* single mutant counterpart. However, *unc-33(mn407)* and *unc-33(e204)* were statistically different from wildtype (*p* < 0.0001). n = 60 animals per strain.

Besides defects in dauer formation, *unc-33* mutants have severely uncoordinated movements (Tsuboi et al., 2005). To study whether the introduction of the *daf-7(e1372)* mutation into *unc-33* mutants rescues uncoordinated locomotion, the movement of all strains was analyzed on two media: NGM plates seeded with *E. coli* OP50 and wells with liquid media. Strains with the *daf-7* mutation were synchronized as dauers, while the other strains without the *daf-7* mutation were synchronized to be at the L3 life stage. Analysis of nematodes’ locomotion on NGM plates showed that the severely uncoordinated locomotion characteristics of *unc-33* mutants was not rescued by introducing the *daf-7(e1372)* mutation (Figure 2A). Statistical evaluations of distance traveled after 10 min revealed no significant difference (*p* > 0.9999) between the single mutants *unc-33(mn407), unc-33(e204),* and double mutants *unc-33(mn407); daf-7(e1372),* and *unc-33(e204); daf-7(e1372).* In contrast, the distance traveled by wildtype nematodes was significantly more than double mutants *unc-33(mn407); daf-7(e1372)* (*p* < 0.0001)*,* and *unc-33(e204); daf-7(e1372)* (*p* < 0.0172).

In addition, locomotion in liquid was assessed in dauers or the L3 life stage of nematodes. Strains were given a 10-s or 15-s acclimation period followed by a 30-s recording of thrashes. The thrashing rate was then multiplied by two to provide a total recorded time of 1 min. In liquid medium, the uncoordinated locomotion of *unc-33* mutants was not rescued either. Double mutants *unc-33(mn407); daf-7(e1372)* and *unc-33(e204); daf-7(e1372)* dauers showed jerky locomotion and diminished frequencies of body bends which were indistinguishable from those quantified for *unc-33* single mutants (Figure 2B). In the case of *daf-7(e1372)* dauers, we noticed that after leaving the animals in liquid media for several minutes, they displayed a different behavior. First, dauers were lethargic in liquid media and then they became active by producing significantly more body bends per minute after the initial 30-s recording of thrashes. This phenomenon of two different behaviors in liquid was not observed in wildtype, *unc-33* mutants, or any of the *unc-33; daf-7* double mutants.

### 3.3 The *daf-7(e1372)* mutation does not restore axonal outgrowth defects in *unc-33* mutants

**FIGURE 3:**
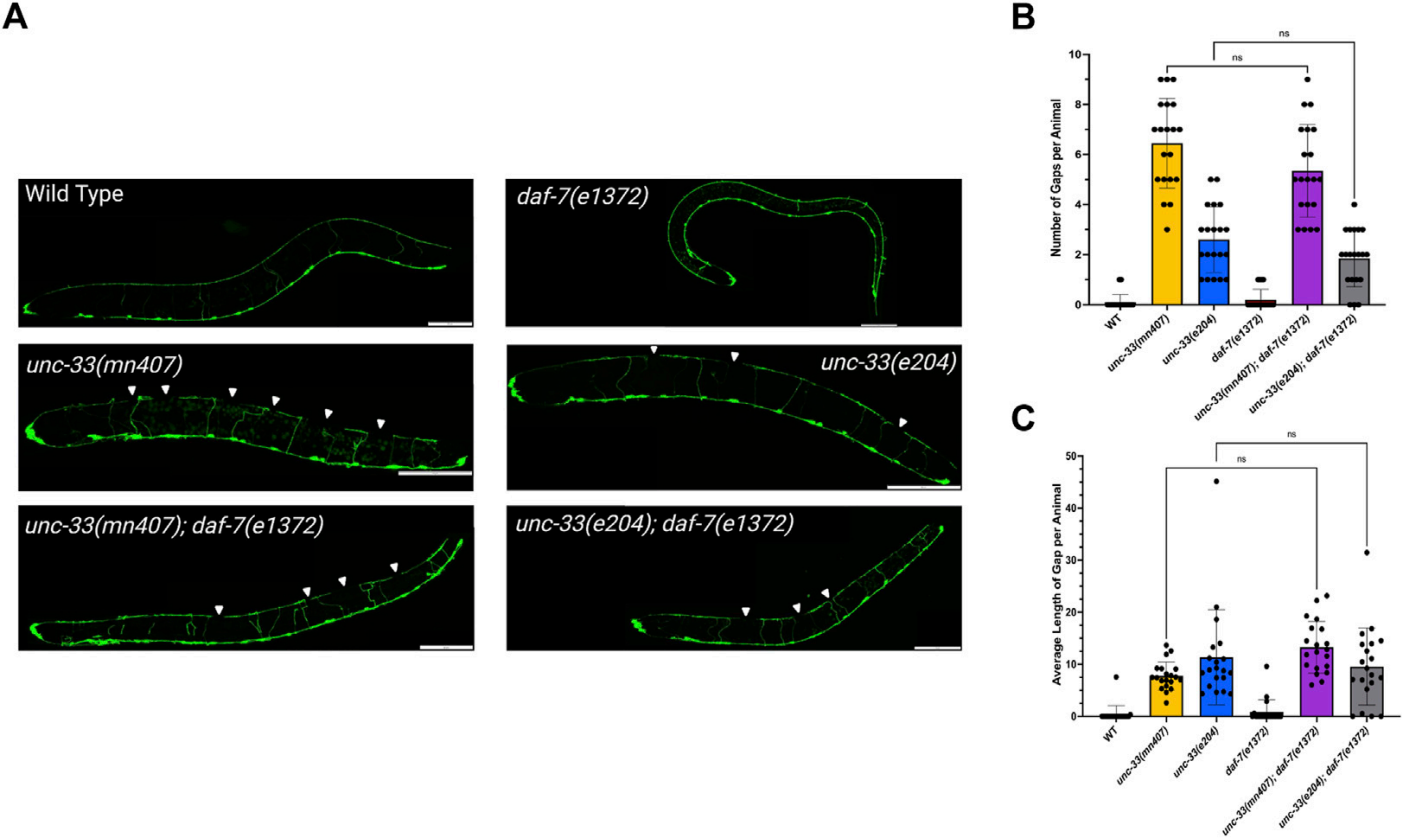
The introduction of *daf-7* mutation into an *unc-33* mutant background does not rescue axonal outgrowth defects seen in *unc-33* mutants **(A)** Images taken of the dorsal (top) and ventral (bottom) nerve cords of all the strains as dauers or the L3 larval stage. White Arrows indicate a gap within the dorsal nerve cord. **(B)** Average gaps within the dorsal nerve cord per animal for each strain. **(C)** Average length of the gaps per animal for each strain. Statistical analysis was performed using the Kruskal–Wallis’s test and the Dunn’s multiple comparisons test. However, *unc-33(mn407)* and *unc-33(e204)* were statistically different from wildtype (*p* < 0.001). n = 20 animals per strain. ns = not significant. Scale bar: 50 µm.

Research has also shown that *unc-33* mutants have defects in axonal outgrowth which result in the uncoordinated movements in *unc-33* mutants (Tsuboi et al., 2005). To assess if the introduction of the *daf-7* mutation to an *unc-33* mutant has the ability to restore axonal elongation, the dorsal nerve cord for each strain was imaged in dauers or the L3 life stage using a transgene that expresses GFP in D-type motor neurons (*juIs76*) (Figure 3A). Multiple images for each individual animal were captured for total visualization of the dorsal nerve cord and then the images were merged together using the Multiple Image Alignment algorithm in cellSens (Olympus). The average number of gaps and the average length of each gap were recorded for each strain. Statistical analysis of the number of gaps showed no significant difference between the *unc-33* single mutants and the *unc-33; daf-7* double mutants (*p* > 0.9999, Figure 3B). Additionally, there was no significant difference in the average length of gap for the *unc-33* single mutants and the *unc-33; daf-7* double mutants further indicating that the introduction of *daf-7* mutation to an *unc-33* mutant background does not restore axonal elongation in dauer *unc-33; daf-7* double mutants (Figure 3C). The lack of restoration of axonal outgrowth in *unc-33; daf-7* double mutants aligns with the previous findings denoting that locomotion is still impaired in *unc-33; daf-7* double mutants (Figure 2).

### 3.4 Adult *unc-33; daf-7* double mutants have defects in locomotion

After analyses revealed that *unc-33; daf-7* double mutants dauers still have defects in locomotion, another study was conducted to determine if adult double mutants recovering from the dauer stage would have the same abnormalities. We evaluated the locomotion of adults that developed after exiting the dauer stage for plate locomotion and locomotion in liquid. Strains with the *daf-7* mutation were selected by synchronizing them at 25°C to promote dauer formation and then shifted to 20°C to induce them to leave dauer diapause to develop into adults. Strains without the *daf-7* mutation were chosen as they entered adulthood. The strains were then placed either on an NGM agar plate or in a liquid medium.

**FIGURE 4:**
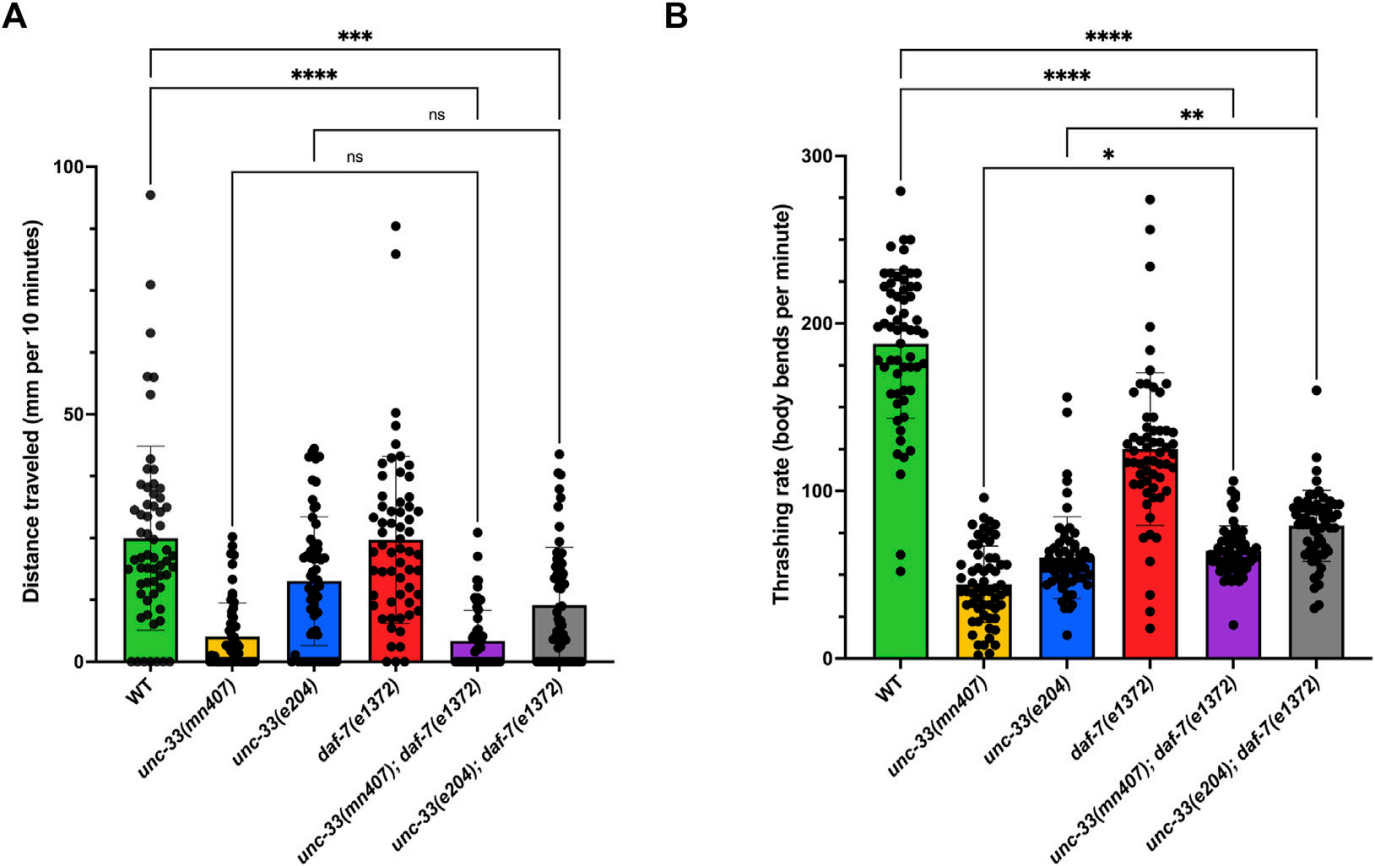
*unc-33; daf-7* mutant adults that were previously dauers show that locomotion defect persists **(A)** Average distance traveled in millimeters per 10 minutes for each strain on a plate seeded with OP50 *E. coli*. **(B)** Average thrashing rate (body bends per minute) for each strain in liquid media. Statistical analyses using Kruskal–Wallis’s tests followed by Dunn’s multiple comparisons show no significant differences (ns) between double mutants *unc-33; daf-7* and their *unc-33* single mutant counterpart. However, *unc-33(mn407)* and *unc-33(e204)* were statistically different from wildtype (*p* < 0.001). n = 60 animals per strain.

Strains that were placed on an NGM agar plate were given a total of 10 minutes to travel on the plate. Images were then taken and quantified using LASX software. The analysis revealed that *unc-33; daf-7* double mutants traveled the same distance as their *unc-33* single mutant counterparts (Figure 4A). For liquid locomotion, results revealed that *unc-33; daf-7* double mutants had partial rescue as denoted by the increase of thrashing rates when compared to the *unc-33* single mutants (*p* < 0.022 for the *unc-33(mn407)* allele and *p* < 0.0036 for *unc-33(e204)*, Figure 4B). However, the locomotion of *unc-33; daf-7* double mutants showed a significantly lower thrashing rate in comparison to wildtype animals (*p* < 0.0001, Figure 4B) which implies the double mutants are severely uncoordinated in locomotion. Together, these results further suggest that the introduction of *daf-7* mutation to an *unc-33* background does not fully restore locomotion defects seen in *unc-33* single mutants as it does for the case of dauer formation defects. However, it is important to note that adult nematodes that recovered from dauer are older than the *unc-33* single mutants that never entered dauer diapause.

## 4 Discussion

**FIGURE 5:**
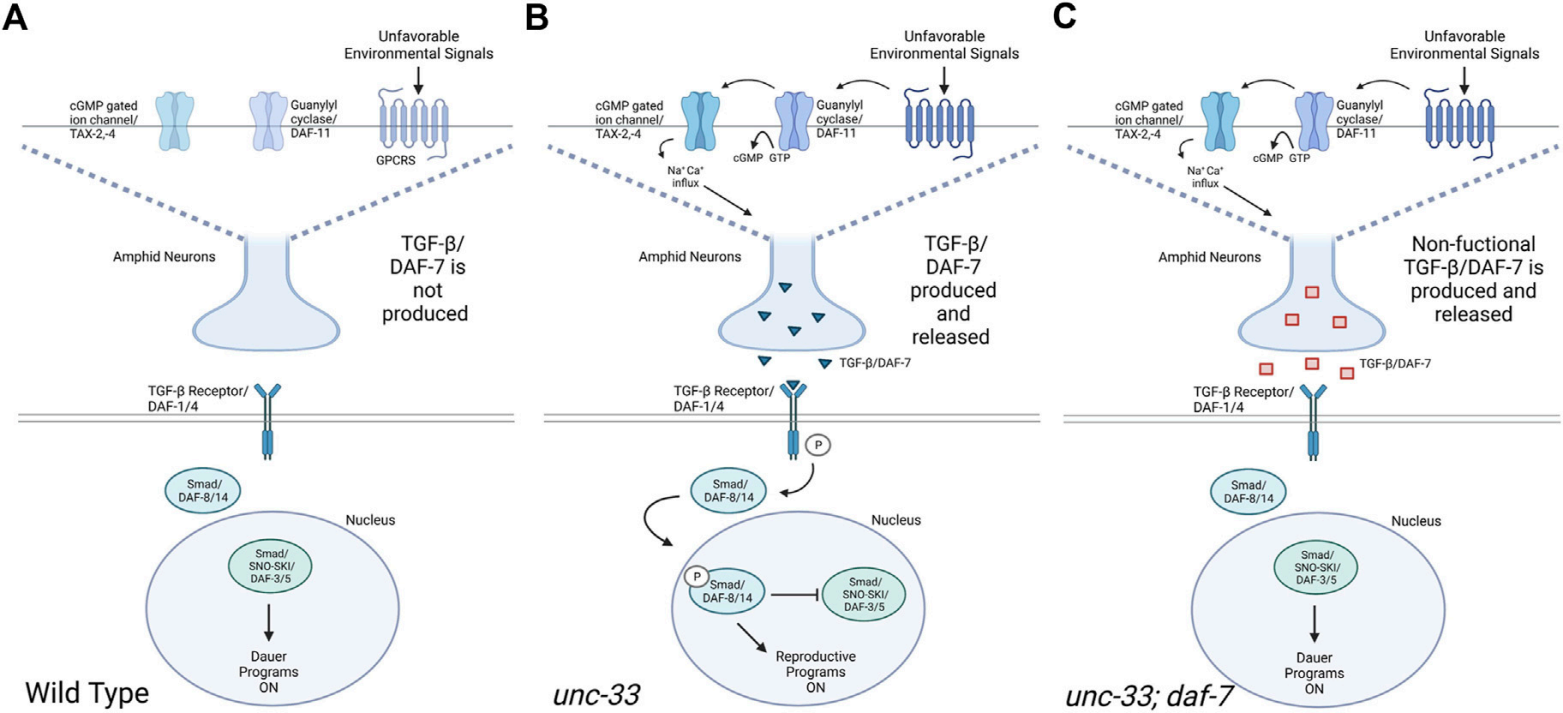
TGFβ/DAF-7-dependent regulation of dauer formation under unfavorable environmental conditions. Schematic models depicting proposed molecular pathways regulating dauer formation in wildtype, *unc-33* mutants, and *unc-33; daf-7* double mutants exposed to unfavorable environmental conditions. **(A)** When unfavorable environmental signals are detected by wildtype animals, GPCRs are not activated, the production TGFβ/DAF-7 is inhibited, and Smad/DAF-8/14 is unable to translocate into the nucleus. In the absence of nuclear DAF-8/14, Smad/SNO-SKI/DAF-3/5 inhibits the biosynthesis of hormones and promotes dauer formation. **(B)** When unfavorable environmental signals are detected by *unc-33* mutants, GPCRs remain active inducing the synthesis of cGMP. High levels of cGMP open cGMP gated ion channels TAX-2,-4 depolarizing amphid neurons which release TGFβ/DAF-7. The ligand TGFβ/DAF-7 binds to the TGFβ receptor which phosphorylates Smad/DAF-8/14. Phosphorylated Smad/DAF-8/14 translocates into the nucleus and inhibits Smad/SNO-SKI/DAF-3/5 which promotes reproductive programs to remain active. **(C)** Similarly, to *unc-33* mutants*,* when *unc-33; daf-7* double mutants are exposed to unfavorable environmental signals, GPCRs remain active leading to the release of the mutant TGFβ/DAF-7. However, the mutant TGFβ/DAF-7 does not bind to the TGFβ receptor, Smad/DAF-8/14 is not phosphorylated rendering Smad/SNO-SKI/DAF-3/5 active, and dauer formation programs ON. Diagram created with BioRender.com.

In *C. elegans,* TGFβ/DAF-7 helps regulate growth and development and allows entry into dauer arrest (Fielenbach and Antebi, 2008; Karp, 2018). Dauer diapause is a developmental stage entered under unfavorable environmental conditions such as overcrowding, lack of resources, and high temperatures, thus enabling *C. elegans* to survive up to several months without feeding (Fielenbach and Antebi, 2008). *C. elegans* interpret environmental cues through chemosensory neurons which initiate neuro-endocrine signaling to influence development and behaviors (Pandey et al., 2021). Previous research has found that *unc-33* mutants are incapable of making dauers (Lopez et al., 2022), however, the mechanism behind this inability to enter dauer diapause is still unknown. Our research shows that dauer formation defects in *unc-33* mutants can be rescued by the introduction of the *daf-7(e1372)* mutation indicating that the inability of *unc-33* mutants to enter dauer diapause could be related to a defect within the DAF-7 pathway in *unc-33* mutants. Studies have shown that UNC-33 is essential for neuronal polarity, microtubule assembly, and formation of axons (Tsuboi et al., 2005). It has been documented that loss of *unc-33* alters microtubule polarity in axon and dendrites, suggesting that UNC-33 regulates microtubule orientation in neuronal processes (Harterink et al., 2018). UNC-33/CRMP2 binds to tubulin heterodimers to promote microtubule assembly and stability (Kodama et al., 2004; He et al., 2020). Research has shown that *unc-33(mn407)* mutants prematurely terminate the axonal process of ASI neurons which would normally extend to the nerve ring (Tsuboi et al., 2005). Additionally, studies of *unc-33(mn407)* mutants found a mislocalization of the sensory chemoreceptor protein ODR-10 from the cilium to axons (Maniar et al., 2012). The neuro-endocrine signal DAF-7 is predominantly expressed in ASI neurons, chemosensory neurons that detect environmental cues and regulate the progression through the life cycle of the nematode (Wang and Levy, 2006). The improper axonal formation of the ASI neurons and the mislocalization of ODR-10 in *unc-33* mutants could explain the defects in the DAF-7 signaling pathway and the inability to form dauers in unfavorable conditions. We postulate that under unfavorable environmental conditions wildtype nematodes inhibit the production and release of DAF-7, activating programs that lead to dauer formation (Figure 5A) (Fielenbach and Antebi, 2008). In contrast, we hypothesize that *unc-33* mutants fail to form dauers under unfavorable environmental conditions because as a cytoskeletal protein it may indirectly interferes with the inhibition of DAF-7 release or may contain defects in channels that regulate membrane potentials in ASI neurons or may affect neurons acting upstream of the ASI neurons (Figure 5B). Therefore, the introduction of the *daf-7(e1372)* mutation into an *unc-33* mutant background restores dauer formation by conditionally inhibiting DAF-7 and impeding the activation of its downstream pathway (Figure 5C).

Besides its inability to regulate dauer diapause, *unc-33* mutants have \numerous defects in axonal outgrowth of sensory and motor neurons as well as uncoordinated movements (Li et al., 1992; Tsuboi et al., 2005). In this study, our findings revealed that the introduction of the *daf-7(e1372)* mutation into *unc-33* mutants does not restore the uncoordinated locomotion in dauer and adult animals. Further analysis of the dorsal nerve cord of *unc-33; daf-7* double mutants demonstrated that axonal outgrowth defects persisted. These findings suggest that knocking out the DAF-7 pathway affects dauer formation but does not impact the restoration of axonal outgrowth or other neuron-specific developmental features impacted by the mutation in the *unc-33* locus. These results also suggest that DAF-7 does not play a significant role in the regulation of axonal outgrowth in *C. elegans* motor neurons. In contrast, expression and axonal localization of UNC-33 was sufficient for restoring coordinated movements and rescuing axonal outgrowth defects in an *unc-33(mn407)* mutant (Tsuboi et al., 2005). Therefore, we conclude that UNC-33 is necessary for proper axonal outgrowth and motility and these defects found in *unc-33* mutants cannot be resolved through the introduction of *daf-7* mutation. However, while the mutation in the *unc-33* locus may lead to neurodevelopmental defects that disrupt chemosensation and dauer regulation, the addition of the *daf-7(e1372)* mutation to an *unc-33* animal is sufficient to conditionally restore dauer formation.

## Data Availability

The datasets presented in this study can be found in online repositories. The names of the repository/repositories and accession number(s) can be found below: https://drive.google.com/drive/folders/1GYokdZ2WRH5PKRBbG19aHgjC_3VGUSt9?usp=share_link.
